# Genital Ulcer Disease: How Worrisome Is It Today? A Status Report from New Delhi, India

**DOI:** 10.1155/2013/203636

**Published:** 2013-04-03

**Authors:** Sumathi Muralidhar, Richa Talwar, Deepa Anil Kumar, Joginder Kumar, Manju Bala, Nilofar Khan, V. Ramesh

**Affiliations:** ^1^Regional STD Teaching, Training & Research Centre, VMMC & Safdarjang Hospital, New Delhi 110029, India; ^2^Department of Community Medicine, Vardhman Mahavir Medical College, Safdarjang Hospital, New Delhi 110029, India; ^3^Department of Microbiology, Faculty of Dentistry, Jamia Millia Islamia University, New Delhi 110025, India

## Abstract

*Background and Objectives*. Genital ulcer diseases represent a diagnostic dilemma, especially in India, where few STI clinics have access to reliable laboratory facility. The changing STI trends require that a correct diagnosis be made in order to institute appropriate treatment and formulate control policies. The objective of this study was to determine recent trends in aetiology of genital ulcers, by using accurate diagnostic tools. * Methods*. Specimens from 90 ulcer patients were processed for dark field microscopy, stained smears, culture for *H. ducreyi*, and real-time PCR. Blood samples were collected for serological tests. *Results*. Prevalence of GUD was 7.45 with mean age at initial sexual experience as 19.2 years. Use of condom with regular and nonregular partners was 19.5% and 42.1%, respectively. Sexual orientation was heterosexual (92.2%) or homosexual (2.2%). There were 8 cases positive for HIV (8.9%). Herpes simplex virus ulcers were the commonest, followed by syphilis and chancroid. There were no cases of donovanosis and LGV. *Conclusions*. A valuable contribution of this study was in validating clinical and syndromic diagnoses of genital ulcers with an accurate aetiological diagnosis. Such reliable data will aid treatment and better define control measures of common agents and help eliminate diseases amenable to elimination, like donovanosis.

## 1. Introduction

Genital ulcer diseases (GUDs) often represent a diagnostic dilemma, especially in developing countries, like India, where few sexually transmitted infection (STI) clinics have access to reliable laboratory facility. The changing trends in STIs make it imperative that a correct diagnosis be made in order to institute appropriate treatment and formulate policies for control. The annual global incidence of GUD exceeds 20 million cases [1]. In 1960s and 1970s, bacterial GUDs were the commonest. By 1980s, with the advent of HIV, the viral GUDs took over [2]. Thus, over time the trends in microorganisms causing GUD have undergone considerable change across the developed world, with bacterial aetiologies giving way to viral causes. Genital herpes has become the most common STD among clinic attendees [3] and the leading cause of genital ulcers worldwide [4]. For viral GUDs, prevention and counseling are more important rather than early diagnosis and treatment, as is the case with bacterial GUDs [2]. 

Often, in GUDs, it is seen that more than one aetiological agent may be found, if careful evaluation is conducted [5]. An accurate diagnosis of a GUD is often not possible when based solely on history and physical examination, because of the lack of sensitivity and specificity of lesions, even in so-called classic cases [6]. To add to the confusion, mixed or multiple infections [7], or a concurrent infection with HIV, can mask the characteristic clinical features of GUD, thus affecting the institution of appropriate therapeutic regimen. Thus, no clinical characteristic is predictive of aetiology of GUD, which underlines the importance of performing a thorough microbiological evaluation [8]. 

In India, the cultural and sociodemographic diversity is considerable, with the result that GUDs vary in presentation according to aetiology, length of clinical course, age, and immunity of the host, leading to diagnostic confusion and unsatisfactory therapeutic results. Therefore, along with a detailed medical history and physical examination, a reliable laboratory support is essential for management of these lesions. This also implies that the syndromic management should not be indistinctly applied to all cases of GUD, without aetiological diagnosis, unless there is no other recourse.

There are very few laboratories in India, catering to the diagnosis of STIs. Fewer still in numbers are the laboratories that perform accurate diagnostic tests like culture techniques and PCR for GUDs. There is significant variability in morphologic presentation of GUDs making the clinical interpretation unreliable when used without confirmatory laboratory tests [9].

There are not many studies from the developing countries, like India, to corroborate such findings, largely due to a lack of reliable laboratory data to back such studies. This study was conceived and carried out with the primary objective of determining the recent trends in the aetiology of GUDs by using newer and more accurate methods of diagnosis, to ensure authenticity of data. It is an attempt to determine the role of the laboratory in GUD case management. It also aims at establishing the utility of existing guidelines and algorithms for the management of GUD and to suggest changes in them based on the findings of the study. 

Diagnosis of GUD by real-time PCR was introduced for the first time in our institution for STI organisms, in addition to the standard culture and serological tests.

## 2. Methods

An aetioepidemiological study was carried out, over a period of one year (from April 1, 2010 to March 31, 2011), as an operational research project under the aegis of National AIDS Control Organization, in Safdarjang Hospital, a tertiary care hospital of New Delhi, India. The project was evaluated and approved by the ethics committee, of the institution. A written, informed consent was taken from each participant at the time of enrolment in the study and sociodemographic characteristics were all enquired into and recorded. For patients who were minors, the consent was obtained from the parent or a responsible adult accompanying the minor, although the history was taken and all procedures were explained to the minor in question as well.

The study population comprised of 1208 male and female STI clinic attendees, of whom 90 patients presented with GUD, as evidenced by disruption of genital mucous membrane or epithelium, and they constituted the study group. Provision was made for enrolling patients referred from targeted intervention clinics run by the nongovernmental organizations (NGOs).

Genital ulcers were examined by STI clinicians and the most likely diagnosis recorded. Demographic characteristics, history of prior antibiotic use, and details of physical examination were also recorded. Material was collected from the genital ulcers as swabs, from the base and edge of the ulcers [10], and processed for various microbiological tests, within minutes, as the clinic and laboratory are located very close to each other. Swabs collected for real-time PCR were placed in Specimen Transport Medium (STM) and frozen at −20°C until further use. Blood samples were collected from all the GUD patients for serological tests. 

 The various tests performed with the specimens collected included the following.

*For Syphilis*. Dark field microscopy was performed, within twenty minutes of collection, on the serous exudates collected from the ulcer, to look for *Treponema pallidum*. A positive dark field microscopy was taken to be positive for syphilis irrespective of the serological test results for syphilis.

Serological tests performed for syphilis are mentioned below. 

A real-time PCR for *T. pallidum* was also performed on ulcer swabs collected in STM.

*For Chancroid*. A swab was collected from the ulcer for smear and stained with Gram's stain, which was then observed under oil immersion objective for presence of pus cells and Gram-negative coccobacilli in clusters or “fish in stream” appearance.

A second swab was streaked on special media (GC agar base-enriched with isovitalex, haemoglobin, and foetal calf serum with antibiotics) and incubated under appropriate conditions.

A third swab was collected and placed in STM for real-time PCR. 

*For Herpes Simplex Virus*. A swab collected from the base of the ulcer was stained by Giemsa's stain to look for typical multinucleated giant cells (MNGCs) indicative of HSV infections.

A second swab was collected and placed in STM for real-time PCR.

The serological test performed for detection of HSV-2 IgM antibodies was ELISA.

*For Donovanosis*. A small piece of tissue from the ulcer was crushed between two glass slides and stained by Giemsa stain to look for the typical morphology of bacteria and “safety pin appearance” indicative of donovanosis.

*For Lymphogranuloma Venereum (LGV)*. Swabs from ulcers were collected for Chlamydia antigen detection by DFA test. 

When indicated, ELISA for Chlamydia antigen detection in serum, was also performed. 

*Serological Tests*. The various serological tests performed with the patient's blood (serum) samples included

- VDRL test;
- TPHA to confirm VDRL reactive samples;
- FTA-Abs—to confirm VDRL reactive samples;
- ELISA for *T. pallidum* to confirm VDRL reactive samples;
- ELISA for HSV-2 IgM antibody detection;
- ELISA/Rapid test/Simple tests—for antibody detection of HIV antibody. This was done after appropriate pre- and post-test counseling and informed patient consent, as required by the National AIDS Control Organization (NACO) guidelines [11].

*Real-time PCR*. The special swabs in the STM kits were used to collect material from genital ulcers. These swabs were processed, DNA was extracted, and real-time PCR performed for *Treponema pallidum*, Herpes simplex virus-1, Herpes simplex virus-2, and *Haemophilus ducreyi*, as per the manufacturer's instructions. The PCR was standardized with appropriate controls provided by the firm that supplied the PCR kits.

A diagnosis of GUD was made when any or many of the above-mentioned tests yielded a positive result.

## 3. Results

A total of 90 cases of GUD were enrolled among 1208 STD clinic attendees over the study period, giving a prevalence of GUD as 7.45. 

Majority of the GUD patients (62.3%) were between 15 and 34 years of age, with 66.7% males and 33.3% females. There were no transgenders. There were only two minors in the study group (aged 12 and 8 years).

The study group consisted of 67.8% married persons and 85.5% literate, above secondary school level. Among the female patients in the study group most of them were homemakers by occupation, while among the male patients, there were office goers, labourers, and students.

The mean age at initial sexual experience was 19.2 years.

Knowledge of STIs and their occurrence was poor, with over 60% being ignorant about the signs and symptoms of STI.

Use of condom with regular partner was 19.5%, while with nonregular partner it was 42.1%.

Sexual orientation of most of the patients in the study group was heterosexual (92.2%), followed by homosexual (2.2%). 

Most of the subjects (52.2%) reported having sex with a regular partner, while 30% had sex with nonregular partners and 7.8% reported having sex with sex worker.

Among the participants, 31 (34.4%) gave a history of previous STI episodes, ranging from ulcers (51.6%), redness/itching to vaginal discharges. Most of them consulted doctors (67.7%) and sought treatment at a government or private clinic. Most of the patients completed the course of treatment advised (77.4%) and claimed to be completely relieved of symptoms (64.5%). However, the number of patients whose partners also sought treatment was low (25.8%).

Of the 90 cases with GUD, 8 were HIV seropositive (8.9%). 

The commonest GUD encountered in the samples from study population was herpes simplex virus—78.9% clinically and 92.2% aetiologically.

Dark field microscopy was performed on 63 cases of GUD, of which 3 were positive (4.76%). Gram's stain smear was performed in 89 samples of which 3 were positive for *H. ducreyi* (3.4%). Giemsa stain was performed on 87 out of the 90 cases of GUD. Of these, 19 (21.84%) were positive for MNGC.

Culture for *H. ducreyi* was put up in 75 cases of GUD, all of which were negative for growth.

Serological tests were performed on blood samples collected from the GUD cases. The results are as shown in Table 1.

Real-time PCR was performed on 90 samples, for the three important organisms causing GUD-*Treponema pallidum, Haemophilus ducreyi* and HSV 1 & 2. 

The comparison of clinical and laboratory diagnosis of all the cases of GUD is shown in Tables 2 and 3.

*Negative for GUD Pathogens*. There were 8 cases (8.89%) (2 females and 6 males) of clinically diagnosed GUD that were negative for all the tests carried out, and hence no aetiological diagnosis could be made. Two of these cases were treated for primary syphilitic chancre and six others for herpes genitalis, as per the clinical findings. One of the herpetic GUD cases also had tinea manuum and tinea unguinum and hence received antifungal agents too.

*Multiple GUD*. There were 10 cases where more than one GUD was diagnosed clinically (11.11%).  Table 4 shows the aetiological diagnosis of these cases.

## 4. Discussion 

The present study revealed that GUDs are a common occurrence in STD clinic attendees (prevalence 7.45%), especially among males (66.7%). Considering the conservative nature of many Indian societies, it is understandable that GUDs are commoner among males because they have easy access and opportunity of seeking multiple sexual partners and practising high risk behavior, in comparison to females. The age group of patients presenting to the STI clinics in our study was predominantly between 15 and 34 years. Although this is in accordance with the age group seen in several other studies [12–15], and an earlier study from this very centre [16], the lower limit of age seems to have fallen in the present study. This may be a significant finding indicating that STIs may be occurring at an earlier age, and that initiation of sexual activity is earlier now than it was then. Also, this is an economically productive age group and hence may have serious repercussions in loss of man hours at work.

The literacy level in the present study was quite high (85.5%) as compared to a previous study on STIs from the same centre (46.5% secondary school or higher) [16]. It was also seen that several of the clinic attendees were seeking information, or clarifications on information, gained from internet, on STIs. This was a new and significant finding during this study, undoubtedly the result of higher literacy rates. Sexual orientation was predominantly heterosexual in this (92.2%) and older studies (97.2%). Homosexuality has made its presence in our study with 2.2%, reflecting a small, but definite change in sexual behaviour.

In most young sexually active patients with GUD, the aetiology is related to an STI, most commonly HSV-1 or 2. The main causative agent of GUD in this study was herpes simplex virus (whether HSV-1 or HSV-2). This is in agreement with other studies in both the developed [17] and developing countries, like Brazil [18] and India [19, 20]. In an earlier report from this very centre, it was shown that syphilis was the commonest GUD observed over a fifteen-year period, followed by chancroid and herpes genitalis [16]. According to other Indian studies, around the same period, HSV emerged as the commonest GUD [21], as has also been observed by O'Farrell et al. [22] and the present study. HSV-GUD increased from 5.7% to 22.4% [16] in an earlier study at this centre. Our study has shown this to have increased further to 78.9% clinically and 92.2% aetiologically, although the previous study reported on the percentage of HSV with reference to all STIs and not just GUDs. HSV persists indefinitely and can be shed for years, and can be reactivated. This is probably why the prevalence of HSV in developing countries is increasing in recent decades [23]. Another important cause for increase in HSV-GUDs is the escalation of the HIV epidemic since mid to late 1990s [24]. 

In a study performed at Kigali, in 1985, the proportions of chancroid, syphilis, and genital herpes diagnosed among patients with GUD were 18%, 28%, and 19%, respectively and 59% of these patients were identified with HIV-1 infection [25]. 

Apart from HSV-2, HSV-1 is increasingly being recognized as an agent for GUD. Most studies do not provide clear-cut data on this because they do not use the tests which differentiate between HSV-1 and 2. Real-time PCR significantly increased HSV-1 and 2 detection in both early (<5 days) and late (>5 days) presentations and in both first and recurrent episodes. Also, the HSV detection by PCR was achieved in less than 4 hours, leading to a significant reduction in time lag with highly reproducible results. In our study HSV-1 was positive in 29 GUD cases (32.2%), either alone (10), with HSV-2 (19) or with *T. pallidum* (1). In the study by Risbud et al. [23], HSV 1 and 2 prevalence was 39% and HSV-1 was the second most frequently diagnosed microorganism with 7.6% by PCR. A recent study in South Africa showed 4.2% of GUDs to be due to HSV-1 [26]. On the other hand, HSV-1 did not seem to be the causative organism of GUD in an older study in Africa [27]. Thus, geographical variations exist and reliable laboratory data can establish this conclusively.

In most countries around the world, syphilis ranks as the second most frequent cause of GUD (2–25%). This was true of our study too, wherein 9 out of 90 cases yielded positive results for syphilis (10%), while the study in Pune showed 10% primary syphilis and 3.3% prevalence [23]. 

Chancroid is encountered very rarely in most developed [28] and much of the developing nations now [16]. In India too the figures seem to be moving downhill, as is evident from our study where* H. ducreyi* was seen in only 3 out of 90 cases (Real-time PCR-1 and by Gram's stain-3). In the Pune study, *H. ducreyi* was the most common cause of GUD with 26% by Multiplex PCR in 1999 [23], while no *H. ducreyi* has been isolated in Australia since 1998 [29]. A study of GUDs in Zambia yielded 0% prevalence of *H. ducreyi*, while HSV-2 (28%), HSV-1 (0.5%), *T. pallidum* (11.5%), and *C. trachomatis* (3%) were all encountered [30]. 

The present study did not yield a single case of donovanosis or LGV (neither clinically nor aetiologically). This situation is true of many other nations across the world where no donovanosis has been reported in several studies [29]. The trend in our centre may be compared with a previous data from this centre over 15 years, when the incidence of LGV decreased from 3.4% in 1990 to 0.2% in 2004 and incidence of donovanosis decreased from 3.9% in 1990 to 0.2% in 2000 [16].

Since all GUDs are sexually transmitted, often more than one aetiological agent may be found if careful evaluation is conducted with reliable laboratory aid [5]. In our study, there were ten cases of GUD (11.11%) wherein more than one aetiological agent was diagnosed. (Table 4), while the Pune study yielded 7% of such infection [23]. A more recent Pune study showed 9% of mixed infections [20]. Also, our study showed that there were 8 cases of GUD in which no causative agent could be established (8.9%) by any of the laboratory methods, although clinically 6 of them were diagnosed as herpetic ulcers and 2 cases diagnosed as syphilitic and were treated as such. This is corroborated by other studies from India and abroad. One Pune study had 34% cases with no causative agent for GUD [23], while another more recent Pune study had 38% of GUD cases where aetiology could not be confirmed [20]. Also, the Canadian guidelines have stated that even after complete diagnostic evaluation, about 25% of patients with GUD have no laboratory confirmed diagnosis [28]. 

The overall sensitivity and specificity of the national syndromic management algorithm for GUD were 68% and 52%, respectively, in the recent Pune study [20], while in the present study the same figures were 92% and 83%, respectively. The positive and negative predictive values were 86% and 90%, respectively. From a public health perspective, it is more important that a diagnostic approach to GUD has a high sensitivity than a high specificity. This is because a low sensitivity means more infected individuals remaining untreated, resulting in complications and the risk of secondary infections, including HIV.

Also, using the national algorithm 52 (42%) cases in the Pune study were clinically misclassified as either herpetic (18 cases) or nonherpetic (34 cases) GUD, resulting in incorrect treatment [20]. In the present study, there were 6 cases that were clinically diagnosed as non-herpetic while aetiologically they were herpetic. There were no cases that were clinically diagnosed as herpetic but aetiologically nonherpetic, which indicates that clinical diagnosis of herpetic ulcers was accurate. There was one case each, wherein clinically a diagnosis of herpetic or nonherpetic ulcer was made, and the aetiological diagnosis yielded both herpetic and nonherpetic aetiologies. There were seven cases that were clinically diagnosed as herpetic (6) or nonherpetic (1) that were aetiologically negative for both.

The HIV seropositive cases in this study were 8 (8.9%), of whom 3 were males and 5 females. All the 8 HIV seropositive cases presented with genital herpes, and PCR of these 8 cases revealed that 3 of them were positive for both HSV-1 and 2. The significance of this lies in the fact that GUDs are known to facilitate the transmission of HIV and HIV, in turn, can alter the presentation and response to treatment of all the organisms causing the GUD [31]. The use of acyclovir for herpetic GUDs is very relevant, as it has been shown to significantly decrease the population of men shedding HIV from their ulcers, as well as the mean ulcer HIV RNA viral load, which suggests a possible benefit in terms of reduction in HIV transmission. A study in Rwanda confirmed a very high rate of HIV infection among patients with GUD (73.2%), while another study in Zambia showed a similar trend (54%) [32]. The clinical picture of genital ulcer syndromes may be particularly severe and prolonged when the patient is HIV positive, thus requiring longer courses of therapy. Recurrence rates after treatment may also be higher [33]. 

Based on surveys performed in South Africa, since 2007, syphilis is generally detected now in less than 10%, and chancroid in less than 1% of GUDs. Therefore, a new GUD flowchart has been devised which gives the option of prescribing antiherpes therapy without the addition of antimicrobial agents for syphilis and chancroid in patients deemed to be at low risk of having acquired an STI [24]. 

## 5. Conclusion

Men and women with GUD are at an increased risk of acquiring and transmitting HIV via a variety of biological mechanisms [34]. This makes it imperative to strengthen measures to control GUDs which in turn would reduce the ability to transmit HIV by decreasing the amount and frequency of HIV shedding.

GUD is often caused by pathogens for which suitable therapies exist, but clinical and laboratory diagnosis may be problematic. There is dire need for rapid and accurate approach to this, as earlier studies have shown [35]. The use of real-time PCR in routine diagnostic settings is limited by concerns over its cost and contamination. But, when validated and used, it is a highly reproducible, rapid, and labour efficient method for HSV detection in genital swabs. This study has demonstrated the potential for this technology to replace conventional culture as a sensitive and rapid means of detecting HSV, especially in a developing country. Also, real-time PCR for HSV allows not only detection of HSV DNA, but also differentiation between HSV-1 and HSV-2 genotypes. According to WHO, recurrences and subclinical shedding are much less frequent for HSV-1 infection than for genital HSV-2 infection [36]. Taken together, these findings indicate that it may be used as an important tool to detect HSV genomes in GUDs [37].

Another valuable contribution of this study is the validation of clinical and syndromic diagnosis of GUDs by making an accurate aetiological (laboratory) diagnosis. Data thus obtained by using reliable laboratory diagnostic methods will aid specific treatment of GUD and better define the prevalence of each microbe among the at-risk population with a view to control the common GUD agents and eliminate those amenable to eradication, like chancroid and donovanosis [29]. Also, the fact that several GUDs that were clinically diagnosed as nonherpetic turned out to be aetiologically herpetic, while the converse was not true, points to a need to revise existing national STI treatment guidelines in India, to include the treatment for herpes simplex virus of all GUD cases. The same finding has also been documented by a study in South India [38].

The link between GUDs and HIV infection provides a strong case for focusing on behaviour-change education and condom promotion on patients with genital ulcers. Thus, monitoring local aetiologies of GUDs provides valuable information that may aid policy changes for their effective treatment. Also, the strengthening of an STI programme management relies heavily on evidence-based interventions which is empowered by studies on establishing aetiological and antimicrobial susceptibility studies.

## Key Messages

- This is the first study validating the clinical and syndromic diagnosis of GUDs with accurate aetiological methods, like culture and real-time PCR.
- Herpes simplex virus 1 & 2 were the commonest causative agents of GUD, followed by syphilis and chancroid.
- In this study, donovanosis and lymphogranuloma venereum were conspicuous by their absence.
- GUDs with multiple aetiologies, that were missed clinically, were picked up by laboratory methods.

## Figures and Tables

**Table 1:** Results of serological tests performed on GUD cases.

Serological test	Number performed	Number reactive/positive	Percentage positive
VDRL	90	9	10%
TPHA	9	9	100%
FTA-Abs	9	9	100%
ELISA-Tp	9	9	100%
ELISA-HSV-2 IgM	44	2	4.54%
ELISA/Rapid—HIV	90	8	8.9%

**Table 2:** Comparison of clinical and laboratory findings in GUDs.

	Clinical Diagnosis	Gram smear (HD)	Giemsa (MNGC)	DFM	HD Culture	VDRL	TPHA + FTA-Abs + ELISA-Tp	ELISA-HSV-2	Lab. diagnosis
Syphilis	6	1	1	3	0	4	4	0	4
HSV	71	1	16	0	0	2	1	2	18
Chancroid	3	0	0	0	0	0	0	0	0
Chancroid + HSV	7	1	1	0	0	1	1	0	1
Syphilis + HSV	3	0	1	0	0	2	2	0	1

Total	90	3	19	3	0	9	8	2	24

Note: HD: *Haemophilus ducreyi*; MNGC: multinucleated giant cell; DFM: dark field microscopy.

**Table 3:** Comparison of clinical diagnosis with real-time PCR results.

Clinical Diagnosis	HSV-1	HSV-2	*T. pallidum* (Tp)	*H. ducreyi* (Hd)	HSV-1 & 2	HSV-2 + Tp + Hd	HSV-2 + Tp	HSV-1 + Tp	HSV-1 & 2 + Tp	PCR diagnosis
Syphilis (6)	0	2	0	0	0	1	2	1	0	4
Herpetic ulcer (71)	8	39	0	0	16	0	1	0	0	64
Chancroid (3)	0	3	0	0	0	0	0	0	0	0
Chancroid + Herpetic (7)	1	5	0	0	0	0	0	0	0	0
Syphilis + Herpetic (3)	0	0	0	0	1	0	1	0	1	2

Total (90)	9	49	0	0	17	1	4	1	1	70

Note: eight samples were negative for all 3 real-time PCR tests.

**Table 4:** Aetiological diagnosis of mixed infections.

Clinical diagnosis	Gram's stain	Giemsa-MNGC	VDRL	TPHA	FTA-Abs	ELISA-Tp	PCR-HSV-1	PCR-HSV-2	PCR-*T. pallidum *
Chancroid + HSV	Neg	Neg	NR	Neg	Neg	Neg	Neg	Neg	Neg
HSV + chancroid	Neg	Neg	Neg	Neg	Neg	Neg	Neg	**Pos**	Neg
HSV + chancroid	Neg	Neg	Neg	Neg	Neg	Neg	Neg	**Pos**	Neg
Syphilis + HSV	Neg	**Pos**	**1 : 2**	**Pos**	**Pos**	**Pos**	Neg	**Pos**	**Pos**
Chancroid + Herpes	Neg	Neg	Neg	Neg	Neg	Neg	Neg	**Pos**	Neg
HSV + Chancroid	Neg	Neg	**1 : 2**	**Pos**	**Pos**	**Pos**	**Pos**	Neg	Neg
HSV + Chancroid	**Pos**	**Pos**	Neg	Neg	Neg	Neg	Neg	**Pos**	Neg
HSV + Chancroid (HIV+)	Neg	Neg	Neg	Neg	Neg	Neg	**Pos**	**Pos**	Neg
HSV + Chancroid (HIV+)	Neg	Neg	Neg	Neg	Neg	Neg	Neg	**Pos**	Neg
Syphilis + HSV	Neg	Neg	**1 : 16**	**Pos**	**Pos**	**Pos**	**Pos**	**Pos**	**Pos**
